# Core Sulphate-Reducing Microorganisms in Metal-Removing Semi-Passive Biochemical Reactors and the Co-Occurrence of Methanogens

**DOI:** 10.3390/microorganisms6010016

**Published:** 2018-02-23

**Authors:** Maryam Rezadehbashi, Susan A. Baldwin

**Affiliations:** Chemical and Biological Engineering, University of British Columbia, 2360 East Mall, Vancouver, BC V6T 1Z3, Canada; maryamdehbashi@gmail.com

**Keywords:** sulphate-reducing microorganisms, sulphate-reducing bacteria, methanogens, biochemical reactors, mine-influenced water, bioremediation, metals, sulphate

## Abstract

Biochemical reactors (BCRs) based on the stimulation of sulphate-reducing microorganisms (SRM) are emerging semi-passive remediation technologies for treatment of mine-influenced water. Their successful removal of metals and sulphate has been proven at the pilot-scale, but little is known about the types of SRM that grow in these systems and whether they are diverse or restricted to particular phylogenetic or taxonomic groups. A phylogenetic study of four established pilot-scale BCRs on three different mine sites compared the diversity of SRM growing in them. The mine sites were geographically distant from each other, nevertheless the BCRs selected for similar SRM types. Clostridia SRM related to *Desulfosporosinus* spp. known to be tolerant to high concentrations of copper were members of the core microbial community. Members of the SRM family Desulfobacteraceae were dominant, particularly those related to *Desulfatirhabdium butyrativorans*. Methanogens were dominant archaea and possibly were present at higher relative abundances than SRM in some BCRs. Both hydrogenotrophic and acetoclastic types were present. There were no strong negative or positive co-occurrence correlations of methanogen and SRM taxa. Knowing which SRM inhabit successfully operating BCRs allows practitioners to target these phylogenetic groups when selecting inoculum for future operations.

## 1. Introduction

Aqueous effluents produced at mine sites may need treatment to remove metals and anions such as sulphate before they can be discharged into the receiving environment [1]. Commonly used chemical treatment involves precipitation of metals by addition of a neutralizing agent such as CaO, Ca(OH)_2_ or CaCO_3_, but this results in production of a large volume of residue that is costly to manage and that might not be stable over the long term [2]. One alternative process using biological sulphate–reduction produces sulphide that reacts with metal cations to precipitate them as metal sulphides. These have a lower solubility product than the metal hydroxides formed during chemical treatment making the final product more stable [3].

Biological treatment processes based on sulphate-reducing microorganisms (SRM) for removal of sulphate and metal ions from mine-influenced water were shown to be effective at the laboratory scale when using defined carbon sources such as ethanol, lactate, and acetate [3,4,5]. In some cases, these studies lead to the development of industrial processes [6]. However, since the carbon source often contributes to the majority of the operating costs of a biological treatment system, it is preferable to use a passive or semi-passive treatment system on the mine site that relies on natural biogeochemical processes within a water-saturated subsurface matrix containing complex carbon sources derived from organic wastes. In these so called biochemical reactors (BCRs), SRM grow on the fermentation by-products from biodegradation of complex, often lignocellulose-rich, organic materials such as forestry or agricultural wastes. If these are available locally, BCRs are more economically favourable for sulphate and metal removal than biological treatment processes requiring defined carbon sources, especially at remote sites, due to their lower construction and operating costs [7,8,9,10]. Because of the complex nature of organic material in these BCRs, a wide diversity of microorganisms are active including cellulose degraders, fermenters, and organisms growing on the fermentation by-products such as SRM and many others [9], with methanogenic Archaea possibly also present completing the final step of carbon mineralization to methane. In addition to being cost effective, the complex organic materials and mixed microbial cultures inside BCRs enable many different mechanisms for metal removal including adsorption, metal reduction, and precipitation of other minerals in addition to sulphides [11]. Of all of the possible mechanisms, SRM are considered one of the most important groups of microbes for successful and sustainable performance of BCRs. Because of this, the environment inside the BCR must be maintained under conditions that are favourable for SRM. These organisms are obligate anaerobes and are only active in the absence of oxygen, or under very low dissolved oxygen concentrations [8]. Low oxygen conditions also promote fermentative processes that provide the low molecular weight carbon sources that are preferred electron donors for SRM. Although acid tolerant SRM are known to exist and be active at low pH, SRM BCRs are most effective at circum-neutral pH [9]. The desirability of passive or semi-passive treatment is often challenged by the effectiveness of the design and operation of the BCR to achieve these favourable conditions for SRM. Additionally, the concentrations of metals and other ions in mine-influenced water that are typically well above natural background levels might be toxic to SRM possibly limiting the types of SRM to those that are metal and saline tolerant. Thus, in order to design, operate and maintain BCRs for reliable removal of metals and sulphate from mine-influenced water it is becoming important to know what types of SRM, and other microorganisms, are present in these bioreactors, and what environmental factors influence their growth and activity.

Known SRM have been classified in several phylogenetic lineages including the bacterial classes Deltaproteobacteria (in orders: Desulfarcules, Desulfobacterales, Desulfovibionales, Desulfurellales, and Desulfuromonadales and Syntrophobacteriales), Clostridia (in the Peptococcaceae and Thermodesulfobiaceae families), Nitrospira (*Thermodesulfovibrio* spp.), and Thermosulfobacteria, as well as in archaea genera *Archaeoglobus*, *Thermocladium*, and *Caldivirga* [12,13]. Early attempts at monitoring sulphate-reducing bioreactors for microbes used culture-based methods [14]. Later, molecular techniques were applied such as quantitative polymerase chain reaction (q-PCR) [15], fluorescence in situ hybridization (FISH) [16], PCR-denaturing gradient gel electrophoresis (DGGE) [17], DNA microarrays [18], and clone library construction [9,15,19]. More recently, next-generation deep sequencing technologies such as Roche 454 Titanium pyrotag, Ion torrent, and Illumina sequencing of the small subunit ribosomal ribonucleic acid (SSU rRNA) genes have proved to be capable of capturing even more of the phylogenetic diversity of entire microbial populations in different ecosystems [20]. The advent of deep SSU rRNA and whole DNA sequencing plus other ‘omics technologies such as metaproteomics (proteins) and meta-metabolomics (metabolites) has opened the door to more expansive monitoring of BCRs, and their metabolic potential can be compared under different conditions. Putative associations and interactions between taxa can be explored. For example, it is not known if SRMs present in a BCR at one site would be different or similar to those at other sites. In the current study, the phylogenetic diversity of SRM in several different field-based BCRs that use complex organics was determined in order to find out if SRM in these systems are restricted to certain phylogenetic lineages or if they are evenly distributed throughout all taxonomic groups. Additionally, the SRM community compositions in the four BCRs were compared with each other to see which phylogenetic lineages were common to all systems. This provided information on which phylogenetic groups of SRM were more adapted to the environment of metal removing BCRs.

Many other types of microbes are present in complex organic BCRs [20]. One group of archaea often found in BCRs are methanogens [15,21]. Like SRM, methanogens thrive in anaerobic environments where they produce methane from CO_2_ and H_2_ (hydrogenotrophic), acetate (acetoclastic) or other compounds such as formate and methyamines. Known methanogens are classified in four Classes: Methanobacteria, Methanomicrobia, Methanococci, and Methanopyri [22]. Sulphate-reducing microbes are thought to outcompete methanogens for electron donors under conditions where sulphate is non-limiting [21,23]. In the absence of sulphate, SRM, which are metabolically diverse, can co-exist syntrophically with methanogens [24]. Previous bioreactor studies with defined carbon sources found that SRM competed with methanogens in high sulphate concentration environments [21,23]. Whereas, SRM and methanogens have been found to co-exist in complex organic BCRs even under conditions where sulphate is present [19,20,25,26]. Both mutualistic and competitive interactions between SRM and methanogens have been observed [27]. On the one hand, methanogens can play a positive role by removing acetate, which is inhibitory to some SRMs under certain conditions [26]. The presence of methanogens did not limit SRM activity when complex organics were used in one study [28]. On the other hand, methanogens might limit SRM growth and activity due to competition for common nutritional sources. In the current study we re-visit the co-occurrence or competition between SRM and methanogens by comparing their relative abundance in four different BCRs and an algae pond used to treat metal-contaminated seepage.

## 2. Materials and Methods

### 2.1. Description of Bioreactors and Sampling

Three sites were selected for this study since they all operate bioreactors treating metal and sulphate containing water using semi-passive treatment technologies (Table 1). Two BCRs and an algae pond at Mine Site 1 treated Cu and Mo containing tailings pond seepage. In one system, water flowed through a series of ponds containing algae into a final subsurface horizontal plug flow BCR packed with manure, woodchips and rocks. Samples were taken using a backhoe excavator from seven different locations in the sludge layer of the final algae pond (Site 1: AP) just prior to the BCR and from within the subsurface matrix of the BCR (Site 1: BCR1). Samples were accessed from both the algae pond and BCR1 on the same day from six random horizontal and vertical locations in each. Another subsurface flow BCR also using wood chips mixed with manure at the same mine site but different location (Site 1: BCR2) was similarly sampled.

Performance of these BCRs at Mine Site 1 is described in a report written by the designers of the treatment process [29]. Data from a previous phylogenetic study of a BCR at another mine site (Site 2: BCR3) were used [20]. This BCR was a subsurface vertical flow anaerobic bioreactor treating Zn, As and sulphate containing seepage and its history has been described elsewhere [10]. The BCR contained pulp and paper mill waste biosolids as the organic matrix. Samples were removed from BCR3 by drilling cores. Data from six random vertical and horizontal locations within BCR3 were used for this study. At the third mine site another subsurface vertical flow system (Site 3: BCR4) treated Cu, Mo, Se and sulphate containing seepage [20]. This BCR differed from the others in that it contained a 3–4 m water cover as opposed to a solid cover of sand and soil as was used for the other BCRs. BCR4 contained a similar organic mixture of woodchips, hay and manure as BCR1 and BCR2. Samples from BCR4 were removed from six random locations in the saturated organic matrix. For comparison, samples were collected also from two locations on Mine Site 1 not impacted by mine-influenced water. Three samples came from the sediments of a natural pond that was used as a source of inoculum for BCR1 and BCR2 (Site 1 IP, Inoculum Pond). Another two samples were taken from the surface of soil several meters away from BCR2. Solid samples collected from all sites were placed immediately in sterile sampling bags (Nasco, Modesto, CA, USA) and frozen using liquid nitrogen. They were stored at −80 °C prior to analysis.

Where possible, environmental parameters temperature, pH, dissolved oxygen and oxidation-reduction potential were measured in the BCR pore water at the time of sampling using a 6820 sonde and a 650 MDS Multiparameter Display System (YSI, Yellow Springs, OH, USA) (Table 1). The operators of the BCRs provided information on the composition of the influents and effluents at the time of sampling.

### 2.2. DNA Extraction, Amplification of SSU rRNA Genes and Pyrosequencing

After homogenization of the sample under liquid N_2_ with a pestle and mortar, DNA was extracted from 0.5 g wet weight using a PowerSoil DNA isolation Kit (MoBio Laboratories Incorporation, Carlsbad, CA, USA, Cat No:12888-100). Isolated community DNA (2 ng μL^−1^) was subjected to polymerase chain reaction (PCR) amplification of variable region V6 to V8 of SSU rRNA of bacteria and archaea by using barcoded primer pair 926f (AAA CTY AAA KGA ATT GAC GG), 1392r (ACG GGC GGT GTG TRC) in an iCycler^®^ (Bio-Rad Laboratories Ltd., Meyerside Drive, ON, Canada) thermocycler under conditions: 95 °C for 3 min; 25 cycles of 95 °C for 30 s, 55 °C for 45 s, 72 °C for 90 s; and 72 °C for 10 min. DNA was purified using the QIAquick^®^ PCR purification kit (Qiagen, Toronto, ON, Canada). DNA concentrations were measured on a NanoDrop^®^ ND-2000 UV-Vis Spectrophotometer (NanoDrop Technologies, Wilmington, DE, USA) and by running 1 µL of the PCR product on a 1% agarose gel. Purified PCR products were sent to the McGill University Innovation Centre (Montréal, QC, Canada) for pyrosequencing using a Roche GS-FLX Titanium Series sequencer. Sequence data for each site were deposited in the sequence read archive (SRA) of the National Center for Biotechnology Information (NCBI) under project accession numbers PRJNA 263922 (BCR1, BCR2, AP, IP, and Soil), PRJNA263922 (BCR3), and PRJNA271309 (BCR4).

### 2.3. Bioinformatics

Raw sequence data generated from pyrosequencing were processed using the QIIME [30] suite of scripts. Sequences were rejected if they: (i) did not perfectly match the primers; (ii) contained ambiguous nucleotides; (iii) were less than 200 bp in length; (iv) had average quality scores below 25; and (v) had homopolymer sequences longer than eight base pairs. The remaining high-quality sequences were clustered using the method of usearch [31] into operational taxonomic units (OTUs) based on three different homology cut-offs: 97%, 94%, and 90% [32] representing species, genus and family level, respectively. Representative sequences for each OTU were assigned taxonomy using BLASTn to the Silva version 132 representative set [22]. OTUs represented by one read only were removed. In order to compare the relative abundance of OTUs between samples, the samples were rarefied to the same sequencing depth of 5242 reads per sample. OTUs for putative SRM and methanogen taxonomic groups were filtered from the rarefied OTU tables. 

Phylogenetic trees with representative sequences for the core 97% homology cut-off OTUs and their nearest neighbours picked by using BLASTn to the NCBI nucleotide database [33] were constructed by trimming the NCBI 16S rRNA sequences to the same region as the pyrotag amplicons, aligning these using MUSCLE version 3.8.31 [34] to the reverse complement of the pyrotag OTU representative sequences followed by tree building with PhyML (nucleotide substitution model HKY, 100 bootstraps) [35]. UniFrac [36] with the default settings in QIIME was used to compare samples based on their SRM and methanogen phylogeny. Co-occurrence of all SRM- and methanogen-related 97% OTUs was assessed using Pearson’s Correlation and construction of a network with highly correlated nodes (greater than 0.8 or less than −0.8, *p*-value less than 0.01) visualized with Cytoscape [37]. Only OTUs represented by more than five reads in total were used for the co-occurrence analysis. To cluster the OTUs and samples in the network, a stochastic spring-embedded algorithm was implemented in Cytoscape [37].

## 3. Results

### 3.1. Taxonomic and Phylogenetic Diversity

#### 3.1.1. Overall Microbial Population Composition

**Figure 1:**
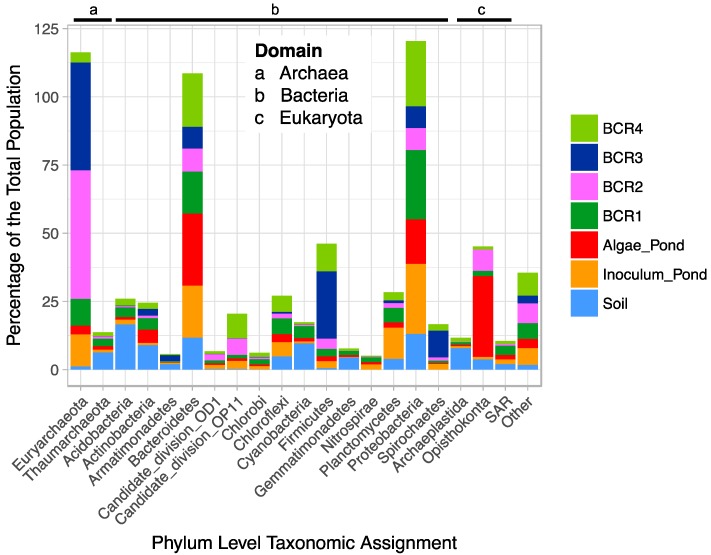
Stacked bar plot of the percentage relative abundance of dominant Phlya in the microbial populations of the BCRs, algae pond, inoculum pond, and soil.

After quality screening, the pyrotag library consisted of 188,712 reads from 35 samples (157,260 reads for the BCRs and algae pond). The V6-V8 primers are universal targeting all Domains, but it is unknown if they amplify targets in all Domains with equal efficiency and coverage. Thus, the between-Domain relative abundances are interpreted with caution. Of the top phyla, representing 95% of the total population from all sites (Figure 1), Euryarchaeota, Bacteroidetes, Firmicutes, and Proteobacteria were most abundant (relatively) in the BCRs.

The Soil microbial community was different from that in the BCRs, as expected, and was dominated by Actinobacteria, Acidobacteria, Cyanobacteria, and Bacteroidetes and Firmicutes. Species within the Bacteriodetes and Firmicutes phyla are associated with organic matter degradation and possibly contained cellulose degraders and fermenters involved in organic matter decomposition in the BCRs. The Algae Pond was dominated by Eukaryota in the phylum Opisthokonta, which were also present in some of the BCRs, as well as Bacteroidetes and Proteobacteria. The species-level taxonomic composition of the populations was not explored in detail as this work focussed on SRM and Methanogens. The species-level (97% identity cut-off OTUs) compositions of the samples were similar within each feature, for the most part, according to Bray-Curtis dissimilarity (Figure S1) suggesting that the samples represent distinct populations within each site that were more distant from other sites. Since microorganisms involved in hydrolysis and fermentation of organic matter are key for production of readily available carbon sources for SRM, it is probable that some of the phylotypes within the taxonomic groups involved in these processes are core to BCRs. In this study we did not explore these, but instead focussed on sub-sets of the core microbiome that included taxonomic groups known to be involved in sulphate reduction and methanogenesis, respectively.

#### 3.1.2. Sulphate-Reducing Microorganisms

OTUs representing putative SRM and methanogens were selected from taxonomic groups known to include species capable of sulphate reduction and methanogenesis, respectively. The Deltaproteobacteria-related OTUs classified within the known SRM orders were selected as were those classified in environmental groups. The latter do not contain any cultured species and therefore it is not known if any of their representatives are capable of sulphate reduction. Some of the clones or sequences classified in these environmental groups have been found in high sulphate environments [20]. The known SRM-related sequences in the bioreactors and algae pond sediment were mostly restricted to Deltaproteobacteria (98% of all SRM-related reads). The most highly represented orders were Desulfobacterales (57%), Syntrophobacterales (11%), Desulfuromonadales (9%), Clostidiales (7%), Desulfovibrionales (3%), and Desulfarculales (1%). *Desulfosporosinus* spp. (in the Clostridiales order and Firmicutes phylum) was the only detected non-Proteobacteria SRM group. Of all of the environmental groups classified in the Deltaproteobacteria class, the Sva0485 group was the most prevalent. Other Deltaproteobacteria environmental groups found in these BCRs included NB1-j, MBNT15 and DTB120.

SRM-related taxonomic groups comprised between 0.3% (BCR3) and 8.3% (BCR4) of all reads in each BCR or the algae pond (Table 2).

**Figure 2:**
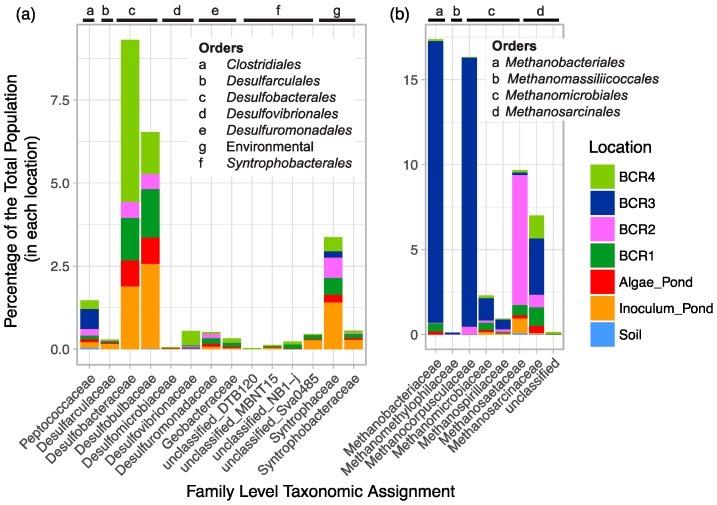
Family-level taxonomic summary diagram of relative abundance of putative (**a**) SRM and (**b**) methanogens in the BCRs and other locations sampled.

Known SRM-related groups were diverse at the Family and Genus levels since there were a total of 62 90% homology cut-off OTUs and 89 94% homology cut-off OTUs. Of all the Family-level classifications, Desulfobacteraceae and Desulfobulbaceae were the most highly represented (Figure 2). Although 7% of the putative SRM reads were classified in the Peptococcaceae family, only a few of these were assigned to known SRM genera. A few Peptococcaceae were assigned to *Cryptanaerobacter*, but most were unclassified. Four Deltaproteobacteria environmental groups were detected. Sequences classified in some of these these environmental groups have been found in other high sulfur environments such as other mine sites [38,39].

Fifty different 97% OTUs accounted for most (80%) of the BCR and algae pond sequences. Both known and unclassified genera were represented. Predominant SRM genus-related OTUs included *Desulfosporosinus*, *Desulfatirhabdium*, *Desulfobacterium*, *Desulfosalsimonas*, *Desulfocapsa*, and *Smithella*. Highly represented novel OTUs were classified in families Desulfobacteraceae and Desulfobulbaceae, and an even more unique OTU could only be classified as uncultured Desulfuromonadales. Of the environmental groups, the sva0081_sediment_group was prevalent in BCR1 and the algae pond.

**Figure 3:**
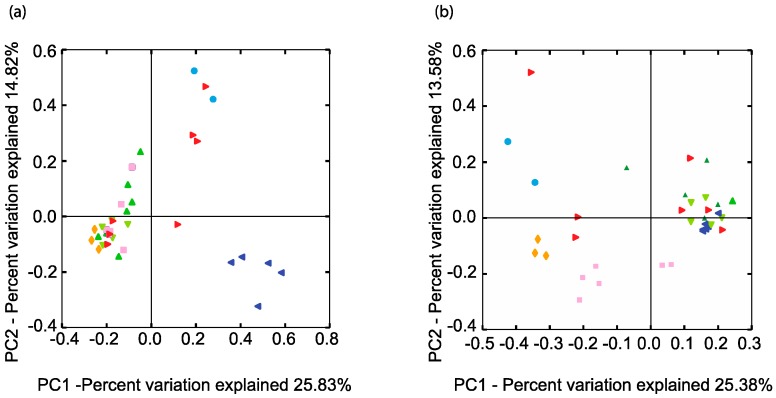
Comparison of the phylogenetic distances of the (**a**) SRM and (**b**) methanogen related communities in the BCRs and other features. Only the two axes of the three-dimensional principal component analysis are shown explaining the most variation. Same colour legend as Figure 1.

A phylogenetic comparison of all the samples with respect to their SRM-related genus-level taxa (94% homology cut-off OTUs) was performed using an unweighted UniFrac analysis [36] (Figure 3). 

In terms of SRM microbial community phylogeny, BCRs 1, 2, and 4 were more similar to each other than the soil or BCR3 (Figure 3). Site 1 BCRs contained very similar SRMs to those found in the pond from which their inoculum came from. The highly predominant unclassified Desulfuromonadales were mostly in BCR1 and the algae pond. The phylogeny of SRM in BCR3 at Site 2 was quite different than that observed at all the other sites. Putative SRM found in this site were restricted to mainly *Desulfosporosinus*, *Desulfovibrio*, and *Smithella*.

**Figure 4:**
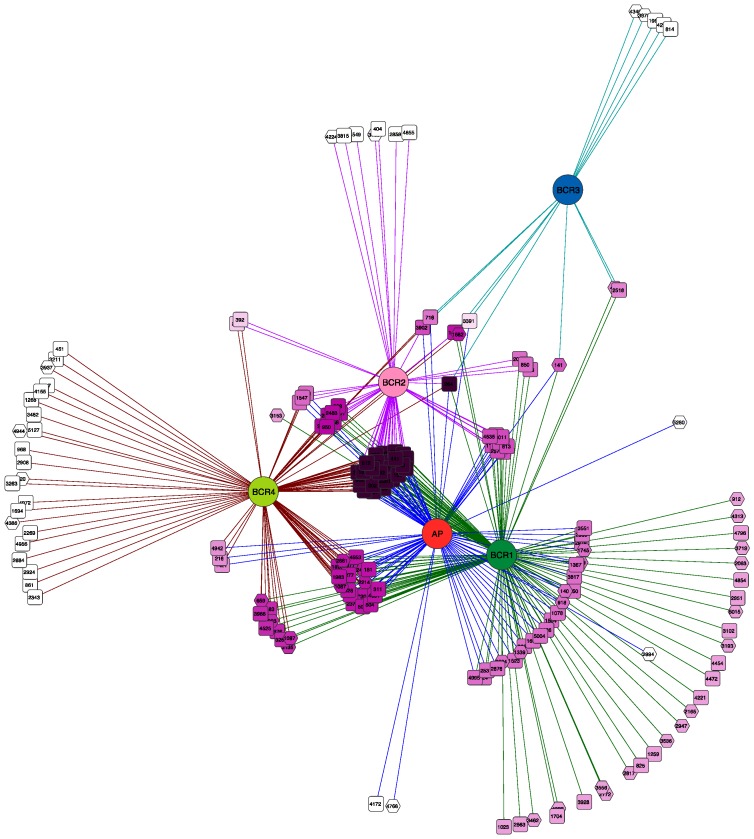
Bipartite network showing SRM operational taxonomic units (OTUs) (square nodes) found in the different BCRs and the algae pond (circular nodes). OTU nodes are connected via lines (edges) to sample nodes in which their sequences were found. Samples cluster together according to their shared OTUs, weighted according to the number of sequences within an OTU. The OTU-nodes are colored according to degree (number of samples that they were in). The darker purple OTU nodes were found in more samples than the OTU nodes with lighter purple color that were restricted to fewer samples. Sample colours are the same as those used in Figure 1, Figure 2 and Figure 3.

The network diagram in Figure 4 displays species-level OTUs that were common to the BCRs and the algae pond, which were defined as those that appeared in more than 50% of samples. The most common OTUs, depicted in dark purple at the centre of the network were found in all samples except BCR3. The latter shared only a few OTUs with a couple of other samples. To learn more about this core group of OTUs, their representative sequences were compared to sequences for cultured species and environmental clone relatives using Blastn to the NCBI refseq and nucleotide databases (Figure S2). Interestingly, the core SRM-related OTUs were not limited to particular phylogenetic lineages, but were distributed across all Orders mostly within Deltaproteobacteria. They were classified in families Desulfobacteraceae, Desulfobulbaceae, Geobacteraceae, Syntrophaceae, and Desulfovibrionaceae. The Firmicutes (Clostridiales) SRM were also represented by an OTU classified in the *Desulfosporosinus* genus. The core SRM community included some OTUs that were also among the most prevalent in some of the BCRs, such as OTU16 classified as *Desulfatirhabdium* (Figure S2).

#### 3.1.3. Methanogens

Methanogen-related sequences comprised 12.7% of all reads from the BCRs and the algae pond indicating that they were overall more prevalent than SRM in these systems. Their taxonomic assignments spanned all known methenogen orders except for those within Methanococci. Taxa were distributed mainly among Methanobacteriales, Methanomicrobiales, and Methanosarcinales and were classified into seven different families (Figure 2). Methanogen-related sequences in BCR2 were taxonomically different from those in the other BCRs since they were mostly assigned to the Methanosaetaceae family (Figure 2 and Figure 3b). Methanobacteraceae and Methanocorpusculaceae were highly represented since they were particularly predominant in BCR3. Interestingly, the methanogenic community in the inoculum pond at Site 1 was quite different from those in the BCRs at the same site. No *Methanobacteria*- or *Methanocorpusculum*-related sequences were detected in the inoculum pond samples. However, a few sequences affiliated with *Methanoregula* and *Methanosaeta* were found. High relative abundance of methanogen-related sequences did not necessarily correlate with greater diversity based on numbers of Family and Species-level OTUs. For example, BCR3 contained by far the greatest proportion of methanogen-related sequences but was represented by fewer 97% homology cut-off OTUs than BCR4, in which methanogens were less represented.

**Figure 5:**
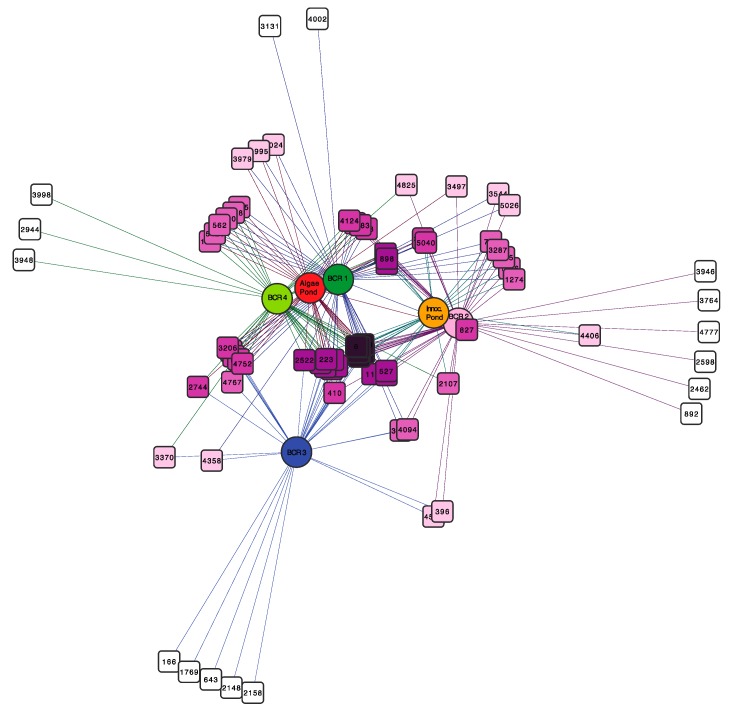
Bipartite network of methanogenic-related OTUs and samples. Legend is the same as in Figure 4.

Some methanogen-related 97% homology cut-off OTUs were found in more than one feature and 19 were common to five or six features (Figure 5). These are methanogens found in many different environments that are closely related to cultured species in all three of the major orders represented in this dataset (Figure S3). *Methanocorpusculum*-related sequences that were very abundant in BCR3 were also found in the other methanogen-rich bioreactor BCR2 but were present in the other features to a much lesser extent. *Methanosaeta concilii*-, *Methanoregula boonei*-, and *Methanosarcina vacuolata*-related OTUs were predominant throughout all BCRs. Methanogen-related OTUs core to these BCRs and the algae pond were also those that were the most prevalent.

### 3.2. RelativeAabundances of SRM- and Methanogen-Related OTUs

Biochemical reactors 1 and 4 had the greatest proportion of reads assigned to SRMs, similar to the inoculation pond at Site 1. Although, BCR4 was located at a different site than BCR1, they both contained organic material of a similar nature. The algae pond and BCRs 2 and 3 all contained proportionally fewer SRM-related sequences with BCR3 being composed of mostly uncultured Deltaproteobacteria and Peptococcaceae. Biochemical reactor 2 and more so BCR3 had larger read counts of methanogen-related sequences than BCR1 and BCR4.

To see if there were any specific SRM or methanogen taxa that co-occurred or were mutually exclusive, Pearson’s correlation analysis was applied pair-wise to the 97% homology cut-off OTU read counts in all the samples. No statistically significant negative correlations were found between any OTUs. Strong positive correlations (greater than 0.8) were found between OTUs within the SRM- and methanogen-related communities, respectively, but not between them (Figure S4). The only observed co-occurrence between SRM- and methanogen-related taxa was for two OTUs classified as Syntrophobacterales (*Syntrophobacter*) and *Methanosaeta*.

## 4. Discussion

### 4.1. SRM Common to BCRs Treating Metal-Containing Effluents

The SRM community common to the metal-rich bioreactors in this study was taxonomically diverse but comprised distinct taxa associated with other metal-rich or saline environments indicating that they might be specialists at surviving under these conditions. Several unclassified groups were prevalent and common revealing that the repertoire of metal-adapted SRM genera is more expansive than previously thought. The only non-Deltaproteobacteria SRM-related taxonomic group classified as *Desulfosporosinus* was closely related to cultured species known to tolerate metal-rich environments. Core *Desulfosporosinus*-classified OTU 297 was closely related to an isolate *Desulfosporosinus* sp. OT (both closely related to *Desulfosporosinus* sp. 5apy (Figure S1)). This isolate was able to survive in copper concentrations in excess (236 mM) of those reported for any other SRM isolated thus far and its genome contained putative copper resistance genes [40]. Other members of this genus are frequently found in metal-rich environments even where the pH is as low as ~2 [41,42,43,44,45,46]. *Desulfosporosinus* species can use electron acceptors other than sulfate [46], such as Fe(III) and arsenic, which were present in some of the BCRs in this study, or nitrate, which is high in mine-influenced water due to the use of explosives. They are metabolically versatile and can use many carbon substrates including polymeric and aromatic compounds [46] such as those possibly present as by-products of organic matter degradation in the BCRs [47]. These organisms might be very important contributors to sulphate-reduction in the BCRs since *Desulfosporosinus* species have been found to contribute to high rates of sulphate-reduction in sediments even though they are rare members of the overall community [48]. Therefore it is not surprising that *Desulfosporosinus*-related SRM are part of the core community for the BCRs of this study.

The most highly represented SRM family, Desulfobacteraceae, comprised genera capable of acetate oxidation. Members of the genus *Desulfatirhabdium* are particularly important in the BCR ecosystem since OTUs assigned to this genus where among the most highly represented and were present in most BCRs forming part of the core. However, little is known about this group as only one isolate has been cultured: *Desulfatirhabdium butyrativorans* gen. nov. (97% homology to OTU 16, Figure S1) isolated from an anaerobic bioreactor treating paper-mill wastewater [49]. There are some indications that *Desulfatirhabdium*-related OTU 16 represents a metal tolerant species since very closely related environmental clones (>99% homology) also came from metal impacted sites [44]. Other BCR core unclassified Desulfobacteraceae OTUs were closely related to environmental clones from oil sands tailings ponds, heavy metal contaminated wetlands, acid mine drainage, and cold lake sediments (based on “isolation site” descriptors in their NCBI reference sequences). Oil sands tailings contain high concentrations of metals in addition to hydrocarbons and are enriched in the deeper anaerobic layers with SRMs and syntrophic communities very similar to those found in the BCRs [50]. OTU 158 was classified as *Desulfosalsimonas*, which includes species isolated from a highly saline lake [51]. Many of the environmental clones assigned to this novel poorly characterized genus came from brine milieus according to the “isolation site” descriptors in their NCBI reference sequences. High salt-water bodies and mine-influenced water have in common elevated total dissolved solids that selects for those organisms able to deal with osmotic stress. 

Core OTUs within the other highly represented SRM family, Desulfobulbaceae, were closely related to cultured species, such as *Desulfobulbus* sp. and *Desulfocapsa* sp., distinguished by their ability to grow on intermediate sulphur compounds [2,52,53]. Sulphide formation through disproportionation of sulphur is a very ancient metabolic process dating back to very early times in the Earth’s history (current estimates are 3.5 billion years ago) [54]. Some of the organisms performing sulphur disproportionation require continuous removal of sulphide, which might be the case in BCRs with high concentrations of metal ions that co-precipitate sulphide. These genera are largely thought to be incomplete oxidizers that produce acetate [55]. Closely related clones come from environments similar to the BCRs such as oil sands tailings and other composting bioreactors treating metal-rich industrial wastes [56]. 

A clade of unclassified Desulfuromonadales play an important role in the BCRs. The core OTUs were classified within environmental groups BVA18 and M20-Pitesti [57]. Because of where these clones were found, there is some indication that these groups are involved with iron reduction concomitant with degradation of aromatic compounds [57]. Since OTUs classified in the Desulfuromonadales order were included in the survey, *Geobacter* spp. closely related to the BCR core OTUs were found in metal contaminated environments. *Geobacter* spp. can reduce sulphur compounds as well as metals [58]. Members of this genus couple acetate oxidation with reduction of many different electron acceptors [59]. *Geobacter* could play an important role in metal remediation in the BCRs due to several specialized capabilities including chemotaxis towards metal ions, direct interspecies electron transfer and the ability to completely oxidize many carbon substrates in extreme metal-rich environments such as radioactive and petrochemical contaminated sites [59].

Syntrophic microorganisms were common to the BCRs, especially those related to *Syntrophus* and *Smithella* species. They were likely involved in obligate or facultative syntrophy with SRM and methanogens. Indeed co-occurrence analysis revealed strong associations between *Smithella*-related OTUs and SRM-related OTUs. *Smithella propionica*, a close relative of the BCR OTUs, is known to be syntrophic with hydrogen utilizing SRMs [60]. The presence of methanogens that can use hydrogen efficiently at low partial pressures can greatly enhance the rate of biodegradation of more recalcitrant carbon compounds that are fermented by bacteria that are syntrophs with methanogens [61]. Syntrophy makes the overall reaction more thermodynamically favourable [61]. A close cultured relative of the BCR *Syntrophus* OTUs, *S. aciditrophicus* SB is a candidate for this type of relationship since its genome contains genes for reverse electron transport and it metabolises fatty and aromatic acids. This could be one explanation for why methanogens become more predominant in highly degraded environments, such as BCR3 [20]. 

Most previous studies on SRM community analysis focussed on laboratory bioreactors and not field-based pilot-scale systems. In laboratory bioreactors, the experimental apparatus can influence the microbial community results [26]. Previous to our study, *Desulfovibrio* species were thought to be the most prevalent SRM in BCRs treating metal-rich water in defined and complex carbon sources [19,25]. The SRM common to the BCRs and other metal-rich sites reveals that a much more diverse community exists of SRM adapted to metals than previously thought. It will be important for future laboratory experiments to use consortia containing these groups as inocula for their bioreactors to see if metal removal efficiency is improved.

### 4.2. Co-Occurrence or Competition with Methanogens

Sulphate-reducing bacteria and methanogens co-exist in these BCRs. In some cases, SRM might be more predominant than methanogens (BCR1 and BCR4), whereas methanogens might dominate over SRM in others (BCR2 and BCR3). Actual more quantitative analysis of the relative abundance of bacteria and archaea still needs to be done since the differences in specificity and sensitivity of the universal V6-V8 primers to members of these two Domains was unknown. Biochemical reactor three was in its sixth year of operation since being recharged with pulp and paper biosolids and there was some evidence for correlation of the presence of methanogens with the degree of degradation of the biosolids [20]. Methanogens appear to have outcompeted SRM in BCR3.

The major SRM (*Desulfobacterium*, *Desulfatirhabdium*, *Desulfobacula*) and methanogen (*Methanosaeta* and *Methanoregula*) taxa represented in the BCRs where SRM and methanogens co-occurred were for those able to use acetate. These bioreactors contain many organisms capable of producing acetate by fermentation of decaying organic matter. There are few microbes able to use acetate and it can accumulate in bioreactors thereby reducing the pH and contributing to inhibition of sulphate-reduction. Acetoclastic SRM mostly belonging to Desulfobacteraceae are desired in BCRs since they couple acetate oxidation with sulphate-reduction. Their presence keeps acetate concentrations low allowing incomplete oxidizing SRM (such as *Desulfovibrio*) to thrive also. *Desulfovibrio* are known to be metal resistant [62] and are often predominant in bioreactors fed with simple carbon compounds such as ethanol [17]. Co-occurrence analysis revealed that complete and incomplete organic carbon oxidizer-SRM were highly associated with each other. On the other hand, in the BCRs where methanogens were much more predominant than SRMs, methanogens without cytochromes (*Methanobacterium*, *Methanocorpusculum*) were prevalent. The metabolism of members of these taxa is restricted to H_2_/CO_2_ or formate. It might be that the availabilities of acetate and hydrogen as electron donors play a role in determining whether SRM and methanogens co-occur or compete, with the possibility of hydrogenotrophic methanogens outcompeting SRM even when sulphate concentrations are high. Some other studies have made similar observations [63,64]. Since the metabolic potential in BCRs treating sulphate- and metal-rich effluents can vary between sulphate-reduction and methanogenesis, factors affecting the predominance of these groups need to be further investigated since it might affect their performance as well as potential greenhouse gas emissions. This work found that SRM were not among the most dominant organisms, with relative abundances mostly less than 10%. Similar findings were made in laboratory BCRs [65]. The dominant microorganisms were those involved in organic matter degradation and types of these might be different between BCRs possibly influencing the reaction kinetics of sulphate-reduction and metal precipitation due to differing abilities to degrade the complex organic material and supply SRM with electron donors. Thus, more work is need to study these groups also. 

## Figures and Tables

**Table 1:** Properties of the biochemical reactors (BCRs), algae pond, inoculum pond and soil on the day of sample collection.

Property	Site 1					Site 2	Site 3
	Algae Pond	BCR1	BCR2	Inoculum Pond	Soil	BCR3	BCR4
Pore water pH	NA	7.8	6.7	NA	NA	5.6–7.5	7.6
Pore water dissolved oxygen (mg/L)	NA	0.08	0.36	NA	NA	0–1.25	3.17
Pore water oxidation-reduction potential (mV reference Ag/AgCl)	NA	−518	−504	NA	NA	less than −112	−21.4
Metals in the influent	Cu; Mo	Cu; Mo	Cu; Mo	NA	NA	Zn; As; Cd	Cu; Mo; Se
Sulfate concentration in the influent	NA	321	448	NA	NA	80–600	450
*Organic materials*	Algae	Wood chips/Manure	Wood chips/Manure	Natural	Natural	Pulp mill biosolids	Wood/Hay/Manure
*Date of commissioning*	1999	1999	2002	Natural	Natural	2002	2010
*Orientation of flow*	NA	Horizontal plug flow	Vertical flow (up or down)	NA	NA	Vertical upflow	Vertical upflow

**Table 2:** Total number of genus-level taxonomic groups, the number of these assigned to putative SRMs and the percentage of putative SRMs in the total population.

Location	Total Number Genus-Level Taxa	Number SRM Genus-Level Taxa	Percentage SRM
BCR1	2376	70	5.5
BCR2	1637	57	2.2
BCR3	662	10	0.3
BCR4	1687	67	8.3
Algae Pond	2037	69	3.3
Inoculum Pond	1122	56	9.0
Soil	668	6	1.0

## Supplementary Materials

The following are available online at http://www.mdpi.com/2076-2607/6/1/16/s1, Figure S1: Hierarchical clustering of the Bray-Curtis dissimilarity of all samples analyzed with all taxonomic groups (all OTUs) using Ward’s Minimum Variance, Figure S2: Maximum likelihood phylogenetic tree of sulphate-reducing microorganisms (SRM) core to the biochemical reactors (BCRs) and algae pond (AP) and their close cultured and environmental relatives. Size of coloured bubbles is proportional to log to the base 10 of the number of reads for each operational taxonomic unit (OTU) in each BCR or the AP. Colours represent the location, Figure S3: Maximum likelihood phylogenetic tree of core methanogens and their close cultured and environmental relatives. Size of coloured bubbles is proportional to log to the base 10 of the number of reads for each OTU in each BCR or the AP. Colours represent the location, Figure S4: Co-occurrence network of SRM and methanogen species-level OTUs. Only those OTUs with greater than or equal to 0.8 Pearson correlation are shown. Nodes represent OTUs and edges Pearson correlation of greater than or equal to 0.8. Colours represent the locations sampled (BCRs, AP, inoculum pond (IP), and soil).

Supplementary File 1
