# Supplementary material for: Core Sulphate-Reducing Microorganisms in Metal-Removing Semi-Passive Biochemical Reactors and the Co-Occurrence of Methanogens
Source: Microorganisms. 2018 Feb 23;6(1):16. doi: 10.3390/microorganisms6010016 (PMC5874630; doi:10.3390/microorganisms6010016)
Supplement: Supplementary File 1 [file microorganisms-06-00016-s001.pdf]

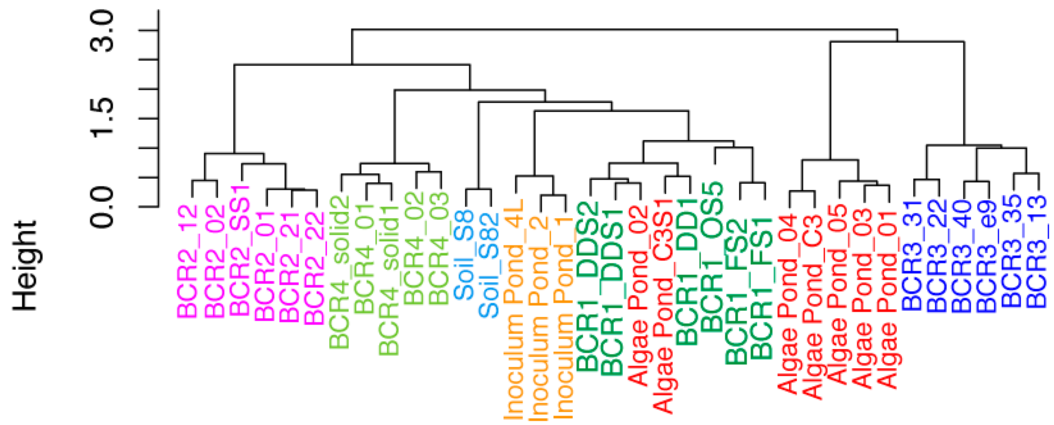

Figure S1. Hierarchical clustering of the Bray-Curtis dissimilarity of all samples analyzed with all taxonomic groups (all OTUs) using Ward’s Minimum Variance.

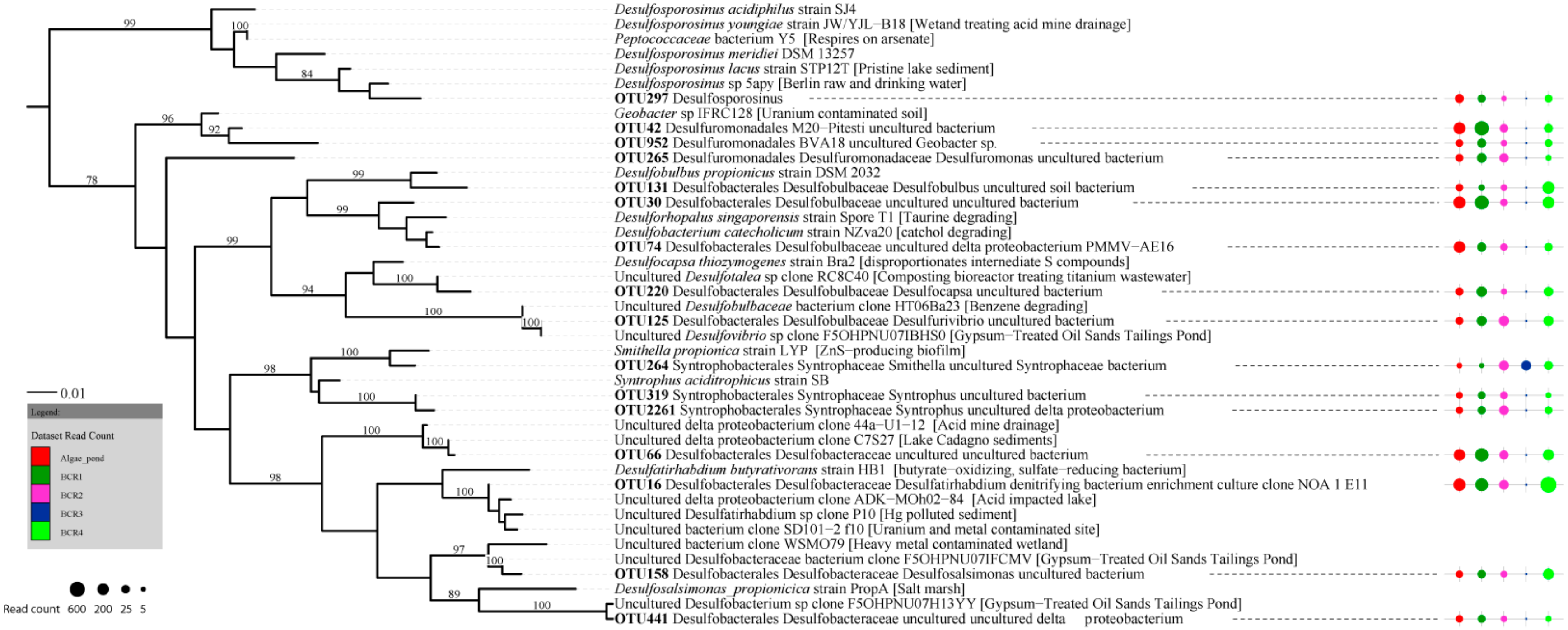

Figure S2. Maximum likelihood phylogenetic tree of sulphate-reducing microorganisms (SRM) core to the biochemical reactors (BCRs) and algae pond (AP) and their close cultured and environmental relatives. Size of coloured bubbles is proportional to log to the base 10 of the number of reads for each operational taxonomic unit (OTU) in each BCR or the AP. Colours represent the location.

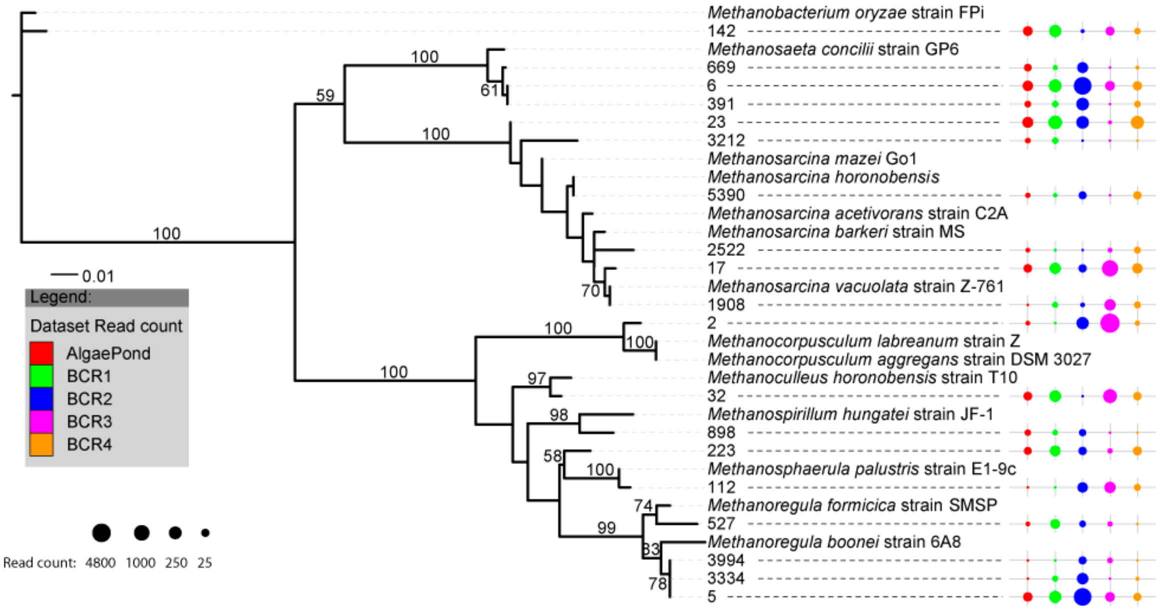

Figure S3. Maximum likelihood phylogenetic tree of core methanogens and their close cultured and environmental relatives. Size of coloured bubbles is proportional to log to the base 10 of the number of reads for each OTU in each BCR or the AP. Colours represent the location.

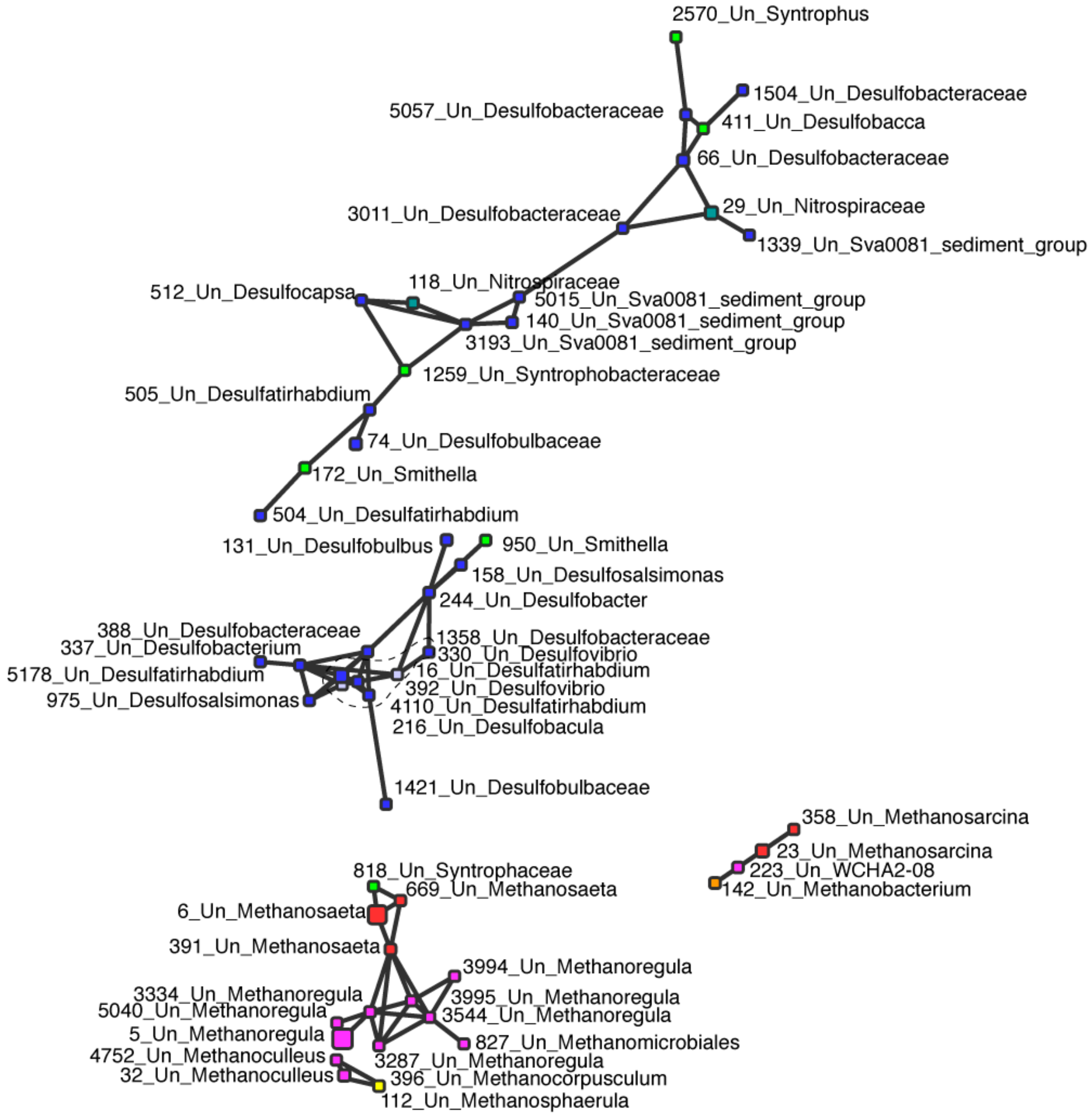

Figure S4. Co-occurrence network of SRM and methanogen species-level OTUs. Only those OTUs with greater than or equal to 0.8 Pearson correlation are shown. Nodes represent OTUs and edges Pearson correlation of greater than or equal to 0.8. Colours represent the locations sampled (BCRs, AP, inoculum pond (IP), and soil).
